# Survival After Intra-Arrest Transport vs On-Scene Cardiopulmonary Resuscitation in Children

**DOI:** 10.1001/jamanetworkopen.2024.11641

**Published:** 2024-05-20

**Authors:** Masashi Okubo, Sho Komukai, Junichi Izawa, SunHee Chung, Ian R. Drennan, Brian E. Grunau, Joshua R. Lupton, Sriram Ramgopal, Thomas D. Rea, Clifton W. Callaway

**Affiliations:** 1 Department of Emergency Medicine, University of Pittsburgh School of Medicine, Pittsburgh, Pennsylvania; 2 Division of Biomedical Statistics, Department of Integrated Medicine, Osaka University Graduate School of Medicine, Osaka, Japan; 3 Department of Internal Medicine, Okinawa Prefectural Chubu Hospital, Okinawa, Japan; 4 Department of Preventive Services, Graduate School of Public Health, Kyoto University, Kyoto, Japan; 5 Department of Critical Care Medicine, Sunnybrook Health Sciences Centre, Toronto, Ontario, Canada; 6 Department of Emergency Medicine, Oregon Health and Science University, Portland; 7 Division of Emergency Medicine, Department of Family and Community Medicine, Temerty Faculty of Medicine, University of Toronto, Toronto, Ontario, Canada; 8 Department of Emergency Medicine, University of British Columbia, Vancouver, British Columbia, Canada; 9 Division of Emergency Medicine, Ann & Robert H. Lurie Children’s Hospital of Chicago, Northwestern University Feinberg School of Medicine, Chicago, Illinois; 10 Department of Medicine, University of Washington, Seattle

## Abstract

**Importance:**

For pediatric out-of-hospital cardiac arrest (OHCA), emergency medical services (EMS) may elect to transport to the hospital during active cardiopulmonary resuscitation (CPR) (ie, intra-arrest transport) or to continue on-scene CPR for the entirety of the resuscitative effort. The comparative effectiveness of these strategies is unclear.

**Objective:**

To evaluate the association between intra-arrest transport compared with continued on-scene CPR and survival after pediatric OHCA, and to determine whether this association differs based on the timing of intra-arrest transport.

**Design, Setting, and Participants:**

This cohort study included pediatric patients aged younger than 18 years with EMS-treated OHCA between December 1, 2005 and June 30, 2015. Data were collected from the Resuscitation Outcomes Consortium Epidemiologic Registry, a prospective 10-site OHCA registry in the US and Canada. Data analysis was performed from May 2022 to February 2024.

**Exposures:**

Intra-arrest transport, defined as an initiation of transport prior to the return of spontaneous circulation, and the interval between EMS arrival and intra-arrest transport.

**Main Outcomes and Measures:**

The primary outcome was survival to hospital discharge. Patients who underwent intra-arrest transport at any given minute after EMS arrival were compared with patients who were at risk of undergoing intra-arrest transport within the same minute using time-dependent propensity scores calculated from patient demographics, arrest characteristics, and EMS interventions. We examined subgroups based on age (<1 year vs ≥1 year).

**Results:**

Of 2854 eligible pediatric patients (median [IQR] age, 1 [0-9] years); 1691 males [59.3%]) who experienced OHCA between December 2005 and June 2015, 1892 children (66.3%) were treated with intra-arrest transport and 962 children (33.7%) received continued on-scene CPR. The median (IQR) time between EMS arrival and intra-arrest transport was 15 (9-22) minutes. In the propensity score–matched cohort (3680 matched cases), there was no significant difference in survival to hospital discharge between the intra-arrest transport group and the continued on-scene CPR group (87 of 1840 patients [4.7%] vs 95 of 1840 patients [5.2%]; risk ratio [RR], 0.81 [95% CI, 0.59-1.10]). Survival to hospital discharge was not modified by the timing of intra-arrest transport (*P* value for the interaction between intra-arrest transport and time to matching = .10). Among patients aged younger than 1 year, intra-arrest transport was associated with lower survival to hospital discharge (RR, 0.52; 95% CI, 0.33-0.83) but there was no association for children aged 1 year or older (RR, 1.22; 95% CI, 0.77-1.93).

**Conclusions and Relevance:**

In this cohort study of a North American OHCA registry, intra-arrest transport compared with continued on-scene CPR was not associated with survival to hospital discharge among children with OHCA. However, intra-arrest transport was associated with a lower likelihood of survival to hospital discharge among children aged younger than 1 year.

## Key Points

**Question:**

Is the transport of children with out-of-hospital cardiac arrest (OHCA) during active cardiopulmonary resuscitation (CPR) associated with a difference in survival to hospital discharge compared with continued on-scene CPR?

**Findings:**

This cohort study of 2854 children who experienced OHCA in the US and Canada found no association between intra-arrest transport and survival to hospital discharge compared with continued on-scene CPR.

**Meaning:**

This cohort study did not find that intra-arrest transport for children with OHCA was associated with improved survival to hospital discharge.

## Teaser

This cohort study examines whether survival differs for children with out-of-hospital cardiac arrest who receive intra-arrest transport compared with continued on-scene cardiopulmonary resuscitation.

## Introduction

Out-of-hospital cardiac arrest (OHCA) is a substantial public health problem among children. Approximately 7000 to 23 000 children experience OHCA each year in the US, with low rates (15%) of surviving to hospital discharge.^1,2,3^ Given the number of lost years per individual, the burden of pediatric OHCA is high.

Emergency medical services (EMS) are a key component of the chain of survival in OHCA management.^4,5,6^ Achieving return of spontaneous circulation (ROSC) is the first step toward long-term recovery. Nonetheless, when patients do not achieve ROSC with initial resuscitation efforts, EMS personnel encounter the challenge of whether to transport to a hospital while continuing cardiopulmonary resuscitation (CPR) during the arrest (intra-arrest transport) or to continue CPR on the scene. A study of adult patients with OHCA showed that intra-arrest transport was associated with an approximately 50% lower likelihood of both survival to and favorable functional outcome at hospital discharge compared with continued on-scene CPR.^7^ In contrast, among pediatric patients with OHCA, there is little evidence to inform clinical decisions regarding the benefit or harm of intra-arrest transport, leading to wide variability in EMS protocols and practices.^8^ A previous observational study reported that an on-scene time (ie, the interval from EMS arrival to either scene departure or termination of resuscitation [TOR]) of 10 to 35 minutes spent by EMS personnel was associated with improved survival after pediatric OHCA compared with an on-scene time of 35 minutes or more, while there was no significant difference in survival between on-scene times of less than 10 minutes and 10 to 35 minutes.^9^ However, that study did not directly compare intra-arrest transport with continued on-scene CPR and did not address resuscitation time bias: that is, a patient with a longer on-scene time likely had a longer resuscitation duration before ROSC.^10^ Better understanding of the optimal transport practice for pediatric OHCA could improve prehospital care systems.

To fill this knowledge gap, we evaluated the association between intra-arrest transport compared with continued on-scene CPR and survival after pediatric OHCA. We also aimed to ascertain whether the association between intra-arrest transport and survival differed depending on the timing of transport.

## Methods

### Study Design and Setting

We performed a retrospective analysis of 2005 to 2015 data from the Resuscitation Outcomes Consortium (ROC) Epidemiologic Registry–Cardiac Arrest (ROC Epistry), a prospective multicenter OHCA registry.^11,12^ The datasets included ROC Epistry 1 and 2 (December 1, 2005, to March 31, 2011) and ROC Epistry 3 (April 1, 2011, to June 30, 2015). Details of the ROC Epistry were previously reported and are provided in the eMethods in Supplement 1.^11,12^ We obtained the publicly available, deidentified ROC Epistry data from the National Heart, Lung, and Blood Institute Biologic Specimen and Data Repository Information Coordinating Center. The institutional review board at the University of Pittsburgh deemed this cohort study exempt from review and the informed patient consent requirement due to the use of publicly available deidentified data. We followed the Strengthening the Reporting of Observational Studies in Epidemiology (STROBE) reporting guideline.

### Study Participants

**Figure 1.**
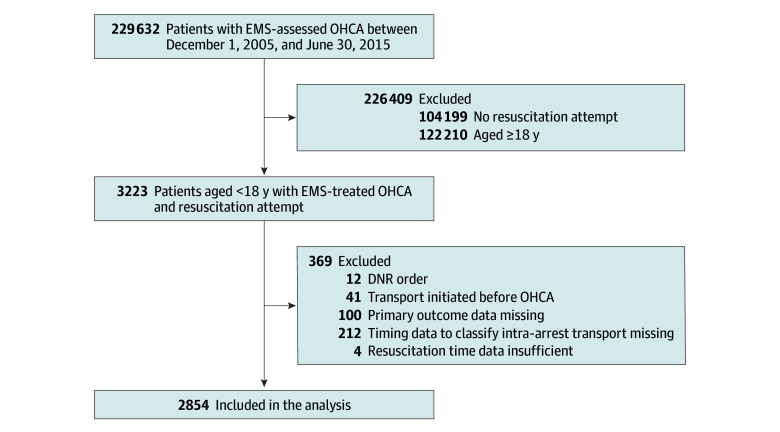
Patient Flow Diagram DNR indicates do-not-resuscitate; EMS, emergency medical services; and OHCA, out-of-hospital cardiac arrest.

We included pediatric patients aged younger than 18 years with EMS-treated nontraumatic OHCA, defined as resuscitation attempts with chest compressions by EMS personnel or shock delivery via external defibrillator by a layperson or EMS personnel. We excluded patients (1) who had TOR because of a preexisting do-not-resuscitate order, (2) for whom transport was initiated prior to cardiac arrest, and (3) who had missing time data to characterize intra-arrest transport or calculate resuscitation time intervals or were missing data on survival to hospital discharge (Figure 1). Resuscitation time intervals included intervals between the 911 call and EMS arrival, between EMS arrival and shock delivery by EMS personnel (if delivered), between EMS arrival and successful advanced airway management (AAM; if performed), between EMS arrival and prehospital ROSC (if achieved), and between EMS arrival and prehospital TOR (if resuscitation was terminated). Shock delivery by a layperson before EMS arrival was included as a time-independent covariate in the propensity score (PS) model (eMethods in Supplement 1). Successful advanced airway placement was determined as the presence of bilateral breath sound when ventilated and the detection of carbon dioxide with a carbon dioxide detector.

### Exposure and Outcomes

The primary exposure was intra-arrest transport, defined as an initiation of transport prior to any episodes of ROSC. The secondary exposure was the interval between EMS arrival and intra-arrest transport.

The primary outcome was survival to hospital discharge. The secondary outcome was survival to hospital admission. Data on survival to hospital admission were available only from April 1, 2011, to June 30, 2015 (ROC Epistry 3) and are reported in the stratified analysis.

### Statistical Analysis

We reported patient demographics, cardiac arrest characteristics, and EMS interventions, stratified according to whether the patient underwent intra-arrest transport or was treated with continued on-scene CPR until ROSC or TOR. We performed time-dependent PS and risk set matching analyses^7,13,14,15,16^ to address resuscitation time bias, a unique limitation of observational studies assessing intra-arrest interventions, such as intra-arrest transport.^10,17^ In the intra-arrest intervention group, patients cannot achieve ROSC before the intra-arrest intervention due to the way the intervention is defined. Therefore, the intra-arrest intervention group tends to have longer resuscitation duration and is biased toward adverse effects (resuscitation time bias), unless the timing of intra-arrest transport is appropriately accounted for.^10,17^ To address this bias, time-dependent PS and risk set matching analyses match patients who underwent intra-arrest transport with patients who were at risk of intra-arrest transport at the same total resuscitation time.

Since survival after pediatric OHCA has improved over time and differs between infants and older children,^3,18^ we stratified the cohort into 4 subgroups, by time period (December 1, 2005-March 31, 2011 [ROC Epistry 1 and 2] vs April 1, 2011-June 30, 2015 [ROC Epistry 3]) and by age (<1 year vs ≥1 year). We carried out the time-dependent PS and risk set matching analyses within each subgroup to avoid matching patients across the subcohorts. We subsequently combined the PS-matched patients and created a total PS-matched cohort.

The time-dependent PS represented the time-varying probability of undergoing intra-arrest transport using a competing risk time-to-event analysis model (Fine-Gray regression model).^13,14,15,16,19,20,21^ In the model, time to intra-arrest transport was the dependent variable, and the time of EMS arrival was set as time 0 because patients were at risk of undergoing intra-arrest transport only after this time point. We used the Fine-Gray model because TOR and ROSC preclude intra-arrest transport, indicating that TOR and ROSC were competing risk events.^22^ Without accounting for the competing risk events, a conventional time-to-event analysis model (eg, Cox regression model) can lead to biased estimates.^22^ We included both time-dependent and time-independent covariates (eTable 1 in Supplement 1). Additional methodological details are provided in the eMethods in Supplement 1.

In the entire PS-matched cohort, to evaluate the association of intra-arrest transport with outcomes, we performed 1:1 risk set matching with replacement using the PS (eMethods in Supplement 1).^10,13,14,15,16,20,21,23,24^ Each patient who underwent intra-arrest transport at any given minute after EMS arrival (exposed patient) was sequentially matched with a patient who was being treated with continued on-scene CPR (ie, was at risk of undergoing intra-arrest transport in that minute) and had a similar PS at the same minute (control patient). These at-risk patients could have subsequently undergone intra-arrest transport after matching because matching should be independent of future events.^10,13,14,15,20,21,23,24^ An at-risk patient could have been matched multiple times as at-risk patients until subsequently undergoing intra-arrest transport (matching with replacement).^13,16,20,23,25^ We set the caliper width for the nearest neighbor matching at 0.2 SDs of the PS in the logit scale.^25,26^ To assess the performance of matching, we calculated a standardized difference for each covariate. Standardized differences of less than 0.25 were regarded as a well-balanced match.^25^

For the primary analysis, we fitted a log link function in generalized estimating equations (GEEs) and estimated risk ratios (RRs) with 95% CIs to assess the association between intra-arrest transport and each outcome.^27^ The RRs represented estimated effect sizes of intra-arrest transport on survival compared with being at risk of undergoing intra-arrest transport. The GEEs were used to address potential within-pair correlation of risk set matching.^13,14,15,20^ In the PS-matched cohort, we used frequency weighting adjustment since some patients in the at-risk group could have been matched more than once as a control.^25^ Next, we conducted stratified analyses in the subgroups by time period (December 1, 2005-March 31, 2011 or April 1, 2011-June 30, 2015) and age group (<1 year or ≥1 year) and assessed the association between intra-arrest transport and outcomes. We tested whether the time period and age group were effect modifiers by including interaction terms between intra-arrest transport and the time period or age group. We considered an effect modifier to be present if the *P* value for the interaction term was significant (*P* < .05).

For the secondary analysis, we fitted 2 models with log link function in GEEs with frequency weighting adjustment to evaluate the timing of intra-arrest transport. One model treated the timing of intra-arrest transport as a categorical variable by 10-minute intervals. The other model treated timing of intra-arrest transport as a continuous variable. In the model with the continuous variable, we included an interaction term between intra-arrest transport and time from EMS arrival to matching, and estimated the RRs of intra-arrest transport at each minute. If *P* for the interaction was <.05, we considered the timing of intra-arrest transport to be associated with the outcome. All tests were 2-sided, and we regarded *P* < .05 as statistically significant. Because of the potential for type I error due to multiple comparisons, findings of the stratified and secondary analyses should be interpreted as exploratory. All statistical analyses were performed with R, version 3.5.1 (R Project for Statistical Computing). Data analysis was performed from May 2022 to February 2024.

## Results

A total of 2854 pediatric patients (median [IQR] age, 1 [0-9] years; 1691 males [59.3%], 1144 females [40.1%], and 19 [0.7%] sex unknown) were eligible for inclusion in this analysis (Table 1). Overall, 1892 patients (66.3%) were treated with intra-arrest transport and 962 (33.7%) received continued on-scene CPR. For patients receiving intra-arrest transport, the median (IQR) time between EMS arrival and intra-arrest transport was 15 (9-22) minutes. In the intra-arrest transport group, 1305 patients (69.0%) received epinephrine and 646 (34.1%) underwent AAM. In the continued on-scene CPR group, 602 patients (62.6%) received epinephrine and 426 (44.3%) underwent AAM.

**Table 1.** Characteristics and Covariates in the Full Cohort

Characteristic or covariate	Patients, No. (%)			Standardized difference^a^
	All (N = 2854)	Intra-arrest transport group (n = 1892)	Continued on-scene CPR group (n = 962)	
Age, median (IQR), y	1 (0-9)	0 (0-5)	2 (0-14)	0.426
Age group				
<1 y	1391 (48.7)	982 (51.9)	409 (42.5)	0.189
≥1 y	1463 (51.3)	910 (48.1)	553 (57.5)	
Sex				
Male	1691 (59.3)	1119 (59.1)	572 (59.5)	0.028
Female	1144 (40.1)	759 (40.1)	385 (40.0)	
Unknown	19 (0.7)	14 (0.7)	5 (0.5)	
Year of OHCA				
December 1, 2005-March 31, 2011 (Epistry 1 and 2)	1516 (53.1)	1035 (54.7)	481 (50.0)	0.094
April 1, 2011-June 30, 2015 (Epistry 3)	1338 (46.9)	857 (45.3)	481 (50.0)	
Time of arrest				
Day (8 am to 8 pm)	1389 (48.7)	939 (49.6)	450 (46.8)	0.113
Night (8 pm to 8 am)	1206 (42.3)	802 (42.4)	404 (42.0)	
Unknown	259 (9.1)	151 (8.0)	108 (11.2)	
OHCA etiology				
Obvious cause	721 (25.3)	411 (21.7)	651 (67.7)	0.238
No obvious cause	2130 (74.6)	1479 (78.2)	310 (32.2)	
Unknown	3 (0.1)	2 (0.1)	1 (0.1)	
OHCA location				
Private location	2573 (90.2)	1725 (91.2)	848 (88.1)	0.114
Public location	279 (9.8)	167 (8.8)	112 (11.6)	
Unknown	2 (0.1)	0 (0)	2 (0.2)	
OCHA witness status				
Layperson	632 (22.1)	428 (22.6)	204 (21.2)	0.166
EMS	100 (3.5)	70 (3.7)	30 (3.1)	
Unwitnessed	1951 (68.4)	1259 (66.5)	692 (71.9)	
Unknown	171 (6.0)	135 (7.1)	36 (3.7)	
Layperson-provided CPR				
Yes	1513 (53.0)	948 (50.1)	565 (58.7)	0.226
No	1160 (40.6)	797 (42.1)	363 (37.7)	
Unknown	181 (6.3)	147 (7.8)	34 (3.5)	
Shock delivery before EMS arrival	21 (0.7)	8 (0.4)	13 (1.4)	0.139
Initial rhythm				
Shockable^b^	171 (6.0)	79 (4.2)	92 (9.6)	0.215
Nonshockable^c^	2379 (83.4)	1611 (85.1)	768 (79.8)	
Unknown	304 (10.7)	202 (10.7)	102 (10.6)	
Interval from 911 call to EMS arrival, median (IQR), min	5 (4-6)	5 (4-6)	4.5 (3.25-6)	0.100
EMS level of care				
ALS first arrival	1317 (46.1)	1014 (53.6)	303 (31.5)	0.505
BLS first arrival before ALS	1455 (51.0)	810 (42.8)	645 (67.0)	
BLS only	78 (2.7)	65 (3.4)	13 (1.4)	
Unknown	4 (0.1)	3 (0.2)	1 (0.1)	
EMS shock delivery	270 (9.5)	146 (7.7)	124 (12.9)	0.171
Interval from EMS arrival to shock delivery, median (IQR), min	7 (4-15)	10 (5-18.75)	5 (3-11)	0.449
Epinephrine administration	1907 (66.8)	1305 (69.0)	602 (62.6)	0.147
Advanced airway placement	1072 (37.6)	646 (34.1)	426 (44.3)	0.209
Interval from EMS arrival to advanced airway placement, median (IQR), min	11 (7-15)	11 (7-15)	10.5 (7-16)	0.036
Interval from EMS arrival to intra-arrest transport, median (IQR), min	NA	15 (9-22)	NA	NA

Abbreviations: ALS, advanced life support; BLS, basic life support; CPR, cardiopulmonary resuscitation; EMS, emergency medical services; Epistry, Resuscitation Outcomes Consortium Epidemiologic Registry–Cardiac Arrest; NA, not applicable; OHCA, out-of-hospital cardiac arrest.

^a^ Standardized difference between intra-arrest and on-scene CPR groups.

^b^ Ventricular fibrillation or pulseless ventricular tachycardia.

^c^ Pulseless electrical activity or asystole.

Using risk set matching, 1840 patients who underwent intra-arrest transport were matched with 1840 patients who were at risk of undergoing intra-arrest transport in the same minute (Table 2). Among those matched as at-risk patients (controls), 1256 patients (68.3%) underwent intra-arrest transport after matching. In the matched cohort, standardized differences were all less than or equal to 0.123, indicating a good postmatching balance. The median (IQR) time between EMS arrival and intra-arrest transport was 15 (9-22) minutes in the intra-arrest transport group and 22 (16-31) minutes in the at-risk group. In the matched cohort, the proportion of patients who survived to hospital discharge did not significantly differ between those who underwent intra-arrest transport (87 of 1840 patients [4.7%]) and those who were at risk of undergoing intra-arrest transport (95 of 1840 patients [5.2%]; relative risk [RR], 0.81 [95% CI, 0.59-1.10]) (Table 3).

**Table 2.** Characteristics and Covariates in Time-Dependent Propensity Score–Matched Cohort

Characteristic or covariate	Patients, No. (%)		Standardized difference
	Intra-arrest transport group (n = 1840)	At-risk of undergoing intra-arrest transport group (n = 1840)	
Age, median (IQR), y	0 (0-4.25)	0 (0-5)	0.010
Age group			
<1 y	957 (52.0)	957 (52.0)	<0.001
≥1 y	883 (48.0)	883 (48.0)	
Sex			
Male	1085 (59.0)	1098 (59.7)	0.026
Female	744 (40.4)	734 (39.9)	
Unknown	11 (0.6)	8 (0.4)	
Year of arrest			
December 1, 2005-March 31, 2011 (Epistry 1 and 2)	1008 (54.8)	1008 (54.8)	<0.001
April 1, 2011-June 30, 2015 (Epistry 3)	832 (45.2)	832 (45.2)	
Time of arrest			
Day (8 am to 8 pm)	909 (49.4)	903 (49.1)	0.011
Night (8 pm to 8 am)	784 (42.6)	785 (42.7)	
Unknown	147 (8.0)	152 (8.3)	
OHCA etiology			
Obvious cause	398 (21.6)	408 (22.2)	0.013
No obvious cause	1440 (78.3)	1430 (77.7)	
Unknown	2 (0.1)	2 (0.1)	
OHCA location			
Private location	1681 (91.4)	1710 (92.9)	0.059
Public location	159 (8.6)	130 (7.1)	
OHCA witness status			
Layperson	410 (22.3)	393 (21.4)	0.035
EMS	64 (3.5)	72 (3.9)	
Unwitnessed	1237 (67.2)	1253 (68.1)	
Unknown	129 (7.0)	122 (6.6)	
Layperson-provided CPR			
Yes	930 (50.5)	952 (51.7)	0.024
No	768 (41.7)	747 (40.6)	
Unknown	142 (7.7)	141 (7.7)	
Shock delivery before EMS arrival	8 (0.4)	7 (0.4)	0.009
Initial rhythm			
Shockable^a^	76 (4.1)	73 (4.0)	0.058
Nonshockable^b^	1568 (85.2)	1602 (87.1)	
Unknown	196 (10.7)	165 (9.0)	
Interval from 911 call to EMS arrival, median (IQR), min	5 (4-6)	5 (4-6)	0.002
EMS level of care			
ALS first arrival	990 (53.8)	970 (52.7)	0.022
BLS first arrival before ALS	791 (43.0)	811 (44.1)	
BLS only	57 (3.1)	57 (3.1)	
Unknown	2 (0.1)	2 (0.1)	
EMS shock delivery	139 (7.6)	157 (8.5)	0.036
Interval from EMS arrival to shock delivery, median (IQR), min	7 (4-13)	7 (5-13)	0.036
Epinephrine administration	1276 (69.3)	1276 (69.3)	0.007
Advanced airway placement	629 (34.2)	738 (40.1)	0.123
Interval from EMS arrival to advanced airway placement, median (IQR), min	10 (7-13)	10 (7-14)	0.089
Interval from EMS arrival to intra-arrest transport, median (IQR), min	15 (9-22)	22 (16-31)	NA

Abbreviations: ALS, advanced life support; BLS, basic life support; CPR, cardiopulmonary resuscitation; EMS, emergency medical services; Epistry, Resuscitation Outcomes Consortium Epidemiologic Registry–Cardiac Arrest; NA, not applicable; OHCA, out-of-hospital cardiac arrest.

^a^ Ventricular fibrillation or pulseless ventricular tachycardia.

^b^ Pulseless electrical activity or asystole.

**Table 3.** Outcomes in Time-Dependent Propensity Score–Matched Cohort

Outcomes	No. of patients with outcome/total No. of patients (%)		Risk ratio (95% CI)
	Intra-arrest transport	At-risk of undergoing intra-arrest transport	
**Primary analysis**			
Survival to hospital discharge	87/1840 (4.7)	95/1840 (5.2)	0.81 (0.59-1.10)
**Stratified analyses**			
December 1, 2005-March 31, 2011 (Epistry 1 and 2)			
Survival to hospital discharge	46/1008 (4.6)	65/1008 (6.4)	0.63 (0.42-0.94)
April 1, 2011-June 30, 2015 (Epistry 3)			
Survival to hospital discharge	41/832 (4.9)	30/832 (3.6)	1.18 (0.72-1.95)
Survival to hospital admission	117/832 (14.1)	91/832 (10.9)	1.26 (0.95-1.68)
Age <1 y			
Survival to hospital discharge	33/957 (3.4)	49/957 (5.1)	0.52 (0.33-0.83)
Age ≥1 y			
Survival to hospital discharge	54/883 (6.1)	46/883 (5.2)	1.22 (0.77-1.93)

In the analyses stratified by time period of the datasets, the proportion of patients who survived to hospital discharge from December 1, 2005, to March 31, 2011, was lower in the intra-arrest transport group than in the at-risk group (RR, 0.63; 95% CI, 0.42-0.94). However, from April 1, 2011, to June 30, 2015, the proportion of patients who survived to hospital discharge did not differ between the intra-arrest transport group and the at-risk group (RR, 1.18; 95% CI, 0.72-1.95) (Table 3). The interaction between intra-arrest transport and the time period was not significant (*P* for interaction = .32). Similarly, the proportion of patients who survived to hospital admission did not differ significantly between the intra-arrest transport group and the at-risk group (RR, 1.26; 95% CI, 0.95-1.68).

In the analyses stratified by age group, there was evidence that the association between intra-arrest transport and survival depended on age (*P* for interaction = .007). The proportion of patients aged younger than 1 year who survived to hospital discharge was lower in the intra-arrest transport group than in the at-risk group (RR, 0.52; 95% CI, 0.33-0.83), while the proportion of patients aged 1 year or older who survived to hospital discharge did not differ between the intra-arrest transport group and the at-risk group (RR, 1.22; 95% CI, 0.77-1.93) (Table 3).

**Figure 2.**
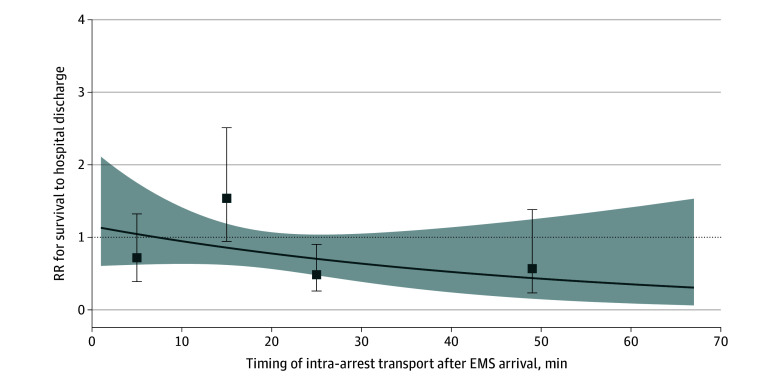
Survival to Hospital Discharge Stratified by Timing of Intra-Arrest Transport Point estimates of the association of intra-arrest transport and survival to hospital discharge (solid line) are reported with 95% CIs (dashed lines), treating timing of intra-arrest transport after emergency medical services (EMS) arrival as a continuous variable. RR indicates risk ratio. Squares indicate point estimates of the association of intra-arrest transport with outcomes with 95% CIs, treating timing as a categorical variable (0 to up to 10 minutes [n = 416], 10 to up to 20 minutes [n = 749], 20 to up to 30 minutes [n = 467], and 30 minutes or longer [n = 208]).

Figure 2 shows the RRs of intra-arrest transport with survival stratified according to the timing of intra-arrest transport compared with the at-risk group. The RRs of intra-arrest transport were 0.72 (95% CI, 0.39-1.32) for 0 to up to 10 minutes, 1.54 (95% CI, 0.94-2.51) for 10 to up to 20 minutes, 0.48 (95% CI, 0.26-0.90) for 20 to up to 30 minutes, and 0.57 (95% CI, 0.23-1.38) for 30 minutes or longer after EMS arrival. The interaction between intra-arrest transport and time to matching was not significant (*P* for interaction = .10). The proportions of patients with survival to hospital discharge in the intra-arrest transport and at-risk groups were 16 of 416 patients (3.8%) vs 27 of 416 patients (6.5%) for 0 to up to 10 minutes, 46 of 749 patients (6.1%) vs 29 of 749 patients (3.9%) for 10 to up to 20 minutes, 17 of 467 patients (3.6%) vs 27 of 467 patients (5.8%) for 20 to up to 30 minutes, and 8 of 208 patients (3.8%) vs 12 of 208 patients (5.7%) for 30 minutes or longer after EMS arrival, respectively.

## Discussion

In this cohort study with time-dependent PS and risk set matching analyses based on a large North American OHCA registry of 2854 children, we observed that intra-arrest transport was not associated with survival to hospital discharge among the overall cohort of children with OHCA compared with continued on-scene CPR. When evaluated by subgroups based on age, however, intra-arrest transport was associated with a lower likelihood of survival to hospital discharge among children aged younger than 1 year. The timing of intra-arrest transport was not associated with survival to hospital discharge.

A recent review of 104 publicly available EMS protocols identified wide variability regarding transport of children with OHCA.^8^ In that review, 38.5% of protocols did not specify when to initiate transport, 32.7% stated to transport after ROSC, and 10.6% stated to transport as soon as possible. These variations highlight the lack of standardized transport practice supported by scientific evidence. Moreover, the findings of potential harm associated with intra-arrest transport among adults^7^ provide a strong rationale to consider how transport strategies may affect pediatric OHCA outcomes.

Few observational studies have investigated the association between on-scene management and patient outcomes after pediatric OHCA. A before-and-after study including 94 children with OHCA reported that favorable functional outcome at hospital discharge improved from 0 of 38 patients (0%) in 2012 and 2013—when EMS personnel provided limited on-scene treatment and focused on rapid transport—to 13 of 56 patients (23%) in 2014 and 2015, after implementation of a protocol in 2014 to facilitate on-scene resuscitative interventions, such as rapid insertion of advanced airway, securing immediate intraosseous access, early epinephrine administration, and tightly controlling the ventilatory rate.^28^ Given that a before-and-after study design cannot account for secular trends of other factors,^29^ it is difficult to make conclusions regarding whether the improved patient outcome is associated with the facilitation of on-scene resuscitative interventions. However, the substantial rise in survivorship is certainly provocative.

A previous retrospective analysis of the ROC data (using some of the same data as this study, spanning 2005-2012) examined the association between scene time (the interval from EMS arrival to either scene departure or TOR) and outcomes. Investigators reported that a scene time of 10 to 35 minutes was associated with improved survival to hospital discharge compared with a scene time of time of more than 35 minutes (adjusted odds ratio [AOR] 0.37; 95% CI, 0.17-0.77), while a scene time of 10 to 35 minutes was not associated with improved survival compared with a scene time of less than 10 minutes (AOR, 0.69; 95% CI, 0.36-1.31).^9^ That study differed from the current investigation in that the previous study investigated the period of time EMS spent at the scene (regardless of whether ROSC occurred early, late, or not at all, and inclusive of prearrest [for EMS-witnessed cases] and post-ROSC time), rather than the period of time that EMS actively provided CPR. It is likely that many cases of extended scene times were due to prolonged resuscitations, which have been associated with unfavorable outcomes.^30^ In contrast, we explicitly compared children who underwent intra-arrest transport with those who received continued on-scene CPR at the same minute after EMS arrival. This comparison addresses the clinically relevant question, “Should this patient undergo intra-arrest transport now?”

Intra-arrest transport was not associated with survival among the overall pediatric population but was associated with a lower likelihood of survival among children aged younger than 1 year. Hence, results of this study would not support a benefit of universal intra-arrest transport for pediatric OHCA, and show a potential unfavorable consequence of intra-arrest transport for children aged younger than 1 year. It should be noted that intra-arrest transport has safety risks to EMS personnel, patients, and the broader communities. Prior research based on National EMS Information System data including 9993 EMS agencies across 49 US states and territories showed that EMS transport with emergency vehicle lights and sirens increased the odds of ambulance accidents (AOR, 2.9 (95% CI, 2.2-3.9) compared with EMS transport without lights and sirens.^31^ Given the safety concerns, the rationale for intra-arrest transport becomes tenuous unless its potential benefits outweigh the associated risks. If more patients undergo continued on-scene CPR, it is likely that fewer patients undergo intra-arrest transport and more patients have prehospital TOR when further resuscitation is deemed futile, which could lead to fewer transports with lights and sirens. However, we must also recognize that EMS personnel face difficulties in terminating resuscitation for pediatric patients. In a survey of 201 paramedics, self-ratings of comfort with TOR on a scale from 1 (very comfortable) to 10 (uncomfortable) found that the median response was 1 for adult and 9 for pediatric patients, highlighting the practical difficulties of prehospital TOR for pediatric OHCA.^32^ Further work is warranted to identify factors associated with these difficulties, and a qualitative approach could characterize domains of such difficulties. The 2022 American Heart Association Resuscitation Guidelines identified the lack of clinical tools to help in the decision to terminate pediatric cardiac arrest resuscitation as one of the crucial knowledge gaps.^6^ Ultimately, when patients are refractory to resuscitation, whether they should undergo TOR or intra-arrest transport is a complex clinical decision affected by patient, family, and EMS factors and available resources, requiring the accumulation of scientific knowledge, guidance from scientific authorities, and shared understanding in a community.

Since our study results showed that intra-arrest transport was associated with a lower likelihood of survival among children aged younger than 1 year, there may be other subgroups of patients who may benefit from either intra-arrest transport or continued on-scene CPR. Due to the limited data resolution and sample size, we were unable to detect such an effect modifier except for the age group. Our findings underscore the need for further research to identify such subgroups, thereby facilitating the tailoring of resuscitation strategies to individual needs.

In contrast to an investigation of adults with OHCA that reported an approximately 50% lower likelihood of survival to hospital discharge associated with intra-arrest transport compared with continued on-scene CPR,^7^ our results show that intra-arrest transport was not associated with survival among pediatric patients with OHCA overall, but was associated with a lower likelihood of survival among children aged younger than 1 year. These 2 studies have a marked difference in sample size. The adult study^7^ included 27 705 patients in the PS-matched cohort, whereas our study included 3680 patients in the PS-matched cohort due to a lower incidence of pediatric OHCA. Given the limited sample size, our results may be underpowered to detect a difference in survival between the intra-arrest transport group and the continued on-scene CPR group. Additionally, the statistical methods were different between the 2 studies. For example, the study in adults included treatment geographic regions as covariates in the PS model and addressed the clustering of patients within each region. Such methodological differences could have contributed to the differences in the results between the 2 studies.

In the full cohort (before PS matching), only 62.6% of patients in the continued on-scene CPR group received epinephrine and 44.3% underwent AAM, suggesting that a limited number of patients underwent these prehospital Advanced Life Support interventions despite continued on-scene CPR. In the intra-arrest transport group, 69.0% received epinephrine and 34.1% underwent AAM. It is possible that a greater proportion of patients with intra-arrest transport subsequently underwent Advanced Life Support interventions after hospital arrival, which might have partially offset the theoretical risk of inadequate CPR metrics during transport.^33,34^ It is also possible that increasing prehospital AAM would not only improve on-scene CPR quality but could improve the safety of transport by facilitating higher-quality oxygenation and ventilation.

### Limitations

This study has limitations. First, intra-arrest transport and time to intra-arrest transport may be associated with EMS systems or geographic regions.^35^ Since information about EMS systems and regions was not available in the publicly available dataset, we were unable to account for such clustering of patients within EMS systems or regions. Additionally, we were not able to adjust for unmeasured confounders, such as patient comorbidities and CPR metrics. Second, it is possible that confounding by indication may have affected our results since the decision to initiate intra-arrest transport was made by EMS personnel, not at random.^36^ A well-powered clinical trial would address these limitations. However, performing such a trial would be challenging due to the sample size limitation and ethical concerns in pediatric OHCA. Alternatively, we conducted an observational study of a large, geographically diverse OHCA dataset using robust statistical methods. Third, the results may not be externally valid at other EMS systems since selected EMS systems were included in ROC based on adherence to performance metrics, ability to conduct trials, and interest in participating in research. Finally, our aim was to evaluate intra-arrest transport compared with on-scene CPR for patients with OHCA without ROSC before the decision for intra-arrest transport was made. Patients experiencing repeat cardiac arrest are outside of our study’s scope.

## Conclusions

In this cohort study of pediatric OHCA in North America, intra-arrest transport was not associated with survival to hospital discharge among children with OHCA compared with continued on-scene CPR. However, intra-arrest transport was associated with a lower likelihood of survival to hospital discharge among children aged younger than 1 year. The timing of intra-arrest transport did not modify its association with survival to hospital discharge.

## Notes

Supplement 1.
eMethods

eReferences

Supplement 2.
Data Sharing Statement
