# Supplementary material for: Survival After Intra-Arrest Transport vs On-Scene Cardiopulmonary Resuscitation in Children
Source: JAMA Netw Open. 2024 May 20;7(5):e2411641. doi: 10.1001/jamanetworkopen.2024.11641 (PMC11107299; doi:10.1001/jamanetworkopen.2024.11641)
Supplement: Supplement 1. — eMethods eReferences [file jamanetwopen-e2411641-s001.pdf]

# Supplemental Online Content

Okubo M, Komukai S, Izawa J, et al. Survival after intra-transport arrest vs on-scene cardiopulmonary resuscitation in children. *JAMA Netw Open*. 2024;7(5):e2411641. doi:10.1001/jamanetworkopen.2024.11641

**eMethods**
**eReferences**

This supplemental material has been provided by the authors to give readers additional information about their work.

**eMethods**

*Resuscitation Outcomes Consortium (ROC) Epidemiologic Registry (Epistry)-Cardiac Arrest*

A case was considered an out-of-hospital cardiac arrest (OHCA), and thus eligible for the registry, if: (1) emergency medical services (EMS) performed chest compressions; (2) if a shock was delivered by an external defibrillator (by layperson or EMS personnel); or, (3) if the patient was identified as pulseless and no treatments were attempted by EMS personnel (cases in this last group were classified as “EMS-untreated”).[1] The ROC was a clinical research network that examined the treatment and subsequent outcomes of patients with OHCA across 10 regional coordinating sites in the United States and Canada.[1,2] The datasets included ROC Epistry 1 & 2 from December 2005 to March 2011 and ROC Epistry 3 from April 2011 to June 2015. To ensure the data integrity, the ROC instituted several quality assurance protocols including periodic training sessions for research teams in data collection and variable definitions, reviews of randomly selected records at each site by the data coordinating center, and logic checks of data element range and consistency in both the online data entry forms and the batch upload process.[1] Moreover, the data coordinating center undertook annual site visits to further review a portion of entered records, data capture process, and site-specific mechanisms for quality assurance.[1] The ROC used several quality assurance plans including periodic training of research teams in data collection and variable definitions, review of randomly selected records at each site by the data coordinating center, and logic checks of data element range and consistency in both the web-based data entry forms and the batch upload process.[1] Additionally, the data coordinating center conducted annual site visits to further review a portion of entered records, data capture process, and site-specific mechanisms for quality assurance.[1]

*Time-dependent Propensity Score*

We calculated the time-dependent propensity score as the time-varying probability of undergoing intra-arrest transport using a competing risk time-to-event analysis model, Fine-Gray regression model.[3-9] In the model, time to intra-arrest transport was the dependent variable, and the time of EMS arrival was the time 0 because patients were at-risk of undergoing intra-arrest transport only after this time point. The time-dependent covariates were shock delivery (if a patient received shock) and successful advanced airway management (AAM) (if a patient received AAM) after EMS arrival. The time-independent covariates were patient age, sex, time of arrest, etiology of arrest, location of arrest, witness status, layperson cardiopulmonary resuscitation (CPR), shock delivery before EMS arrival, initial rhythm, 911 call-to-EMS arrival interval, EMS level of care (advanced life support [ALS] capable EMS personnel arrival before basic life support [BLS] EMS personnel, BLS personnel before ALS, or BLS personnel only] , and epinephrine administration. We used spline functions (B-spline) for continuous variables (age and EMS response time). We chose these covariates *a priori* based on their association with survival from prior knowledge, biologic plausibility, and adequate ascertainment within the dataset.[10-15] We included the first prehospital return of spontaneous circulation (ROSC) and termination of resuscitation (TOR) before intra-arrest transport as competing risk events in the model because intra-arrest transport never occurred after ROSC or TOR except cases with re-arrest after ROSC (i.e., informative censoring events). ROSC and TOR were strongly associated with low likelihoods of intra-arrest transport, which indicated that ROSC and TOR should be treated as competing risk events, not noninformative censoring events.[3-5,7,8]

*Risk-set Matching with Replacement*

We used matching with replacement for two reasons. Firstly, matching with replacement can often decrease bias because controls that are similar to many treated patients can be used multiple times, which is particularly helpful in settings where there are few controls.[16] Secondly, the order in which the exposed patients are matched does not matter—without replacement, the patients who had intra-arrest transport later would not be able to find appropriate control patients since majority of at-risk patients would be matched at an earlier phase.[16]

eReferences


1. Morrison LJ, Nichol G, Rea TD, et al. Rationale, development and implementation of the Resuscitation Outcomes Consortium Epistry-Cardiac Arrest. *Resuscitation.* 2008;78(2):161-169.
2. Davis DP, Garberson LA, Andrusiek DL, et al. A descriptive analysis of Emergency Medical Service Systems participating in the Resuscitation Outcomes Consortium (ROC) network. *Prehosp Emerg Care.* 2007;11(4):369-382.
3. Izawa J, Komukai S, Gibo K, et al. Pre-hospital advanced airway management for adults with out-of-hospital cardiac arrest: nationwide cohort study. *BMJ.* 2019;364:l430.
4. Matsuyama T, Komukai S, Izawa J, et al. Pre-Hospital Administration of Epinephrine in Pediatric Patients With Out-of-Hospital Cardiac Arrest. *J Am Coll Cardiol.* 2020;75(2):194-204.
5. Okubo M, Komukai S, Izawa J, et al. Prehospital advanced airway management for paediatric patients with out-of-hospital cardiac arrest: A nationwide cohort study. *Resuscitation.* 2019;145:175-184.
6. Beyersmann J, Schumacher M. Time-dependent covariates in the proportional subdistribution hazards model for competing risks. *Biostatistics.* 2008;9(4):765-776.
7. Matsuyama T, Komukai S, Izawa J, et al. Epinephrine administration for adult out-of-hospital cardiac arrest patients with refractory shockable rhythm: time-dependent propensity score-sequential matching analysis from a nationwide population-based registry. *Eur Heart J Cardiovasc Pharmacother.* 2021.
8. Amoako J, Komukai S, Izawa J, Callaway CW, Okubo M. Evaluation of Use of Epinephrine and Time to First Dose and Outcomes in Pediatric Patients With Out-of-Hospital Cardiac Arrest. *JAMA Netw Open.* 2023;6(3):e235187.
9. Lu B. Propensity score matching with time-dependent covariates. *Biometrics.* 2005;61(3):721-728.
10. de Caen AR, Maconochie IK, Aickin R, et al. Part 6: Pediatric Basic Life Support and Pediatric Advanced Life Support: 2015 International Consensus on Cardiopulmonary Resuscitation and Emergency Cardiovascular Care Science With Treatment Recommendations. *Circulation.* 2015;132(16 Suppl 1):S177-203.
11. Fink EL, Prince DK, Kaltman JR, et al. Unchanged pediatric out-of-hospital cardiac arrest incidence and survival rates with regional variation in North America. *Resuscitation.* 2016;107:121-128.

12. Jayaram N, McNally B, Tang F, Chan PS. Survival After Out-of-Hospital Cardiac Arrest in Children. *J Am Heart Assoc.* 2015;4(10):e002122.
13. Maconochie IK, Aickin R, Hazinski MF, et al. Pediatric Life Support: 2020 International Consensus on Cardiopulmonary Resuscitation and Emergency Cardiovascular Care Science With Treatment Recommendations. *Circulation.* 2020;142(16_suppl_1):S140-S184.
14. Naim MY, Burke RV, McNally BF, et al. Association of Bystander Cardiopulmonary Resuscitation With Overall and Neurologically Favorable Survival After Pediatric Out-of-Hospital Cardiac Arrest in the United States: A Report From the Cardiac Arrest Registry to Enhance Survival Surveillance Registry. *JAMA Pediatr.* 2017;171(2):133-141.
15. Topjian AA, Raymond TT, Atkins D, et al. Part 4: Pediatric Basic and Advanced Life Support: 2020 American Heart Association Guidelines for Cardiopulmonary Resuscitation and Emergency Cardiovascular Care. *Circulation.* 2020;142(16_suppl_2):S469-S523.
16. Stuart EA. Matching methods for causal inference: A review and a look forward. *Stat Sci.* 2010;25(1):1-21.
