# Supplementary material for: Survival After Intra-Arrest Transport vs On-Scene Cardiopulmonary Resuscitation in Children
Source: JAMA Netw Open. 2024 May 20;7(5):e2411641. doi: 10.1001/jamanetworkopen.2024.11641 (PMC11107299; doi:10.1001/jamanetworkopen.2024.11641)
Supplement: Supplement 2. — Data Sharing Statement [file jamanetwopen-e2411641-s002.pdf]

Data Sharing Statement

Data
Data available: Yes
Data types: Deidentified participant data, Data dictionary
How to access data: https://biolincc.nhlbi.nih.gov/home/
When available: With publication

Supporting Documents
Document types: None

Additional Information
Who can access the data: Anyone can request the data from the NHLBI using the link above.
Types of analyses: Per National Heart, Lung and Blood Institute (NHLBI) Biologic Specimen and Data Repository Information Coordinating Center.
Mechanisms of data availability: Mechanisms of data availability: National Heart, Lung and Blood Institute (NHLBI) Biologic Specimen and Data Repository Information Coordinating Center will review the application
